# Androstenediol Reduces Demyelination-Induced Axonopathy in the Rat Corpus Callosum: Impact on Microglial Polarization

**DOI:** 10.3389/fncel.2017.00049

**Published:** 2017-02-23

**Authors:** Samah Kalakh, Abdeslam Mouihate

**Affiliations:** Department of Physiology, Health Sciences Centre, Faculty of Medicine, Kuwait UniversityKuwait City, Kuwait

**Keywords:** axonal spheroids, neurosteroids, focal demyelination, iNOS, arginase-1

## Abstract

**Aims**: We have previously shown that the neurosteroid androstenediol (ADIOL) promotes remyelination following gliotoxin-induced demyelination. However, the impact of this ADIOL on axonal recovery is not yet known. In the present study, we investigated the impact of ADIOL on axonal integrity following a focal demyelination in the corpus callosum.

**Methods**: A 2 μl solution of either ethidium bromide (EB; 0.04%) or pyrogen-free saline were stereotaxically injected into the corpus callosum of Sprague Dawley rats. Each of these two rat groups was divided into two subgroups and received daily subcutaneous injections of either ADIOL (5 mg/kg) or vehicle. The brains were collected at 2, 7 and 14 days post-stereotaxic injection. Immunofluorescent staining was used to explore the impact of ADIOL on axonal integrity (neurofilament (NF)-M) and microglial activation (ionized calcium binding adapter molecule 1, Iba1). The inducible nitric oxide synthase (iNOS) and arginase-1 (arg-1), two major markers of microglial polarization towards the proinflammatory M1 and the regulatory M2 phenotypes respectively, were monitored using western blot.

**Results**: ADIOL increased the density of NF fibers and decreased the extent of axonal damage in the vicinity of the demyelination lesion. ADIOL-induced decrease in axonal damage was manifested by decreased number of axonal spheroids at both 2 and 7 days post-demyelination insult. This reduced axonopathy was associated with decreased expression of iNOS and enhanced expression of arg-1 during the acute phase.

**Conclusion**: These data strongly suggest that ADIOL reduces demyelination-induced axonal damage, likely by dampening the local inflammatory response in the white matter and shifting microglial polarization towards a reparative mode.

## Introduction

Myelin is a lipid membrane that enwraps a large portion of axons within the nervous system. It enhances the speed of propagation of nerve impulses and provides axons with trophic support (Franklin et al., 2012). Within the central nervous system, this myelin sheath is produced by oligodendrocytes (Nave, 2010a). The pathological loss of myelin (demyelination) leads to inefficient transmission of nerve impulses along axons such as seen in diseases like multiple sclerosis (MS; Franklin and Gallo, 2014). MS is a disease characterized by the development of a wide range of disabilities depending on the area of the central nervous system affected (Crawford et al., 2013). These deficits include sensory, motor, autonomic and cognitive dysfunctions (Procaccini et al., 2015). Demyelinated axons are deprived from trophic support provided by the myelinating oligodendrocytes (Crawford et al., 2013). The demyelination process triggers a local inflammatory response driven largely by microglia (Franklin and Gallo, 2014). The lack of trophic support and the accompanying inflammatory response predispose axons to damage following demyelination (Trapp and Stys, 2009; Stadelmann et al., 2011). Axonal injury (axonopathy) involves disruption in axonal cytoarchitecture and deficits in transport along the affected axons, which lead to the development of axonal swellings (Garay et al., 2009). Ultimately, damaged axons are broken down into condensed spheroids and degenerate subsequently (Trapp and Stys, 2009; Stadelmann et al., 2011). Axonopathy is considered a key determinant of MS disease severity (Peterson and Fujinami, 2007). For example, exploration of postmortem MS brains revealed multiple axonal transections and irregularities in the disease-active areas (Trapp and Stys, 2009; Nave, 2010b). At the end-stage of MS, the loss of axons in the corpus callosum is estimated to be ~70% (Trapp and Stys, 2009). Hence, limiting axonal damage can potentially reduce the severity of the disease.

Microglia, the major immune competent cells of the nervous system, undergo activation in response to demyelination injury (Franklin and Gallo, 2014). Activated microglia become highly mitotic and adopt morphological changes manifested by large perikarya and small processes (Mouihate, 2014). In addition to these morphological changes, activated microglia acquire one of two distinct phenotypes known as M1 and M2 phenotypes (Miron and Franklin, 2014). M1 microglia synthesize and release pro-inflammatory cytokines such as interleukin (IL)-1β, IL-6, tumor necrosis factor-α (TNF-α), which promote the proliferation and the recruitment of oligodendrocytes precursor cells (OPCs) to the site of demyelination (Arnett et al., 2001; Erta et al., 2012). M2 microglia on the other hand produce anti-inflammatory cytokines including IL-4, IL-10 and transforming growth factor-β (TGF-β), which enhance the maturation of OPCs into myelinating oligodendrocytes (Miron and Franklin, 2014). Morphological distinction exists between M1 and M2 microglia. While M1 microglia show a round shape with no apparent processes, the M2 microglial cells appear more elongated and show short processes (Zhang et al., 2014), it is still unclear how the M1/M2 polarization of microglia impact axonal injury following demyelination.

We have previously shown that the dehydroepiandrosterone metabolite known as 5-androsten-3β, 17β-diol (androstenediol (ADIOL)), promotes the process of remyelination following ethidium bromide (EB)-induced demyelination in the corpus callosum of male rats (Kalakh and Mouihate, 2015). In this study, we extend our exploration to the potential effect of ADIOL on axonal recovery following demyelination. Here we give evidence that ADIOL reduces axonopathy following EB-induced demyelination. These protective effects were associated with a shift in microglial polarization from M1 to M2 phenotype at the early stage of demyelination-induced inflammation.

## Materials and Methods

### Animals

Adult male Sprague Dawley rats (250–270 g) were bred in the Animal Resources Centre in Kuwait University. Rats were pair-housed and left in a room with the temperature set to 22°C under a 12-h light-dark cycle (light is on from 7 a.m. until 7 p.m.). The rats had access to pelleted chow and water *ad libitum*. This study and experimental procedures were approved by the Animal Research Ethics committee at the Health Sciences Centre/Kuwait University.

### Demyelination Induction

Induction of focal demyelination in rat corpus callosum was performed as previously described (Kalakh and Mouihate, 2015). Briefly, rats were anesthetized by an intraperitoneal (i.p.) injection of a mixture of ketamine (50 mg/Kg, i.p.) and xylazine (5 mg/Kg, i.p.). Animals were then fixed on the stereotaxic frame and the following coordinates were used to target the corpus callosum: bregma: 0 mm, antero-lateral: 2 mm, dorso-ventral: 3.4 mm (Paxinos and Watson, 2006). To induce demyelination, 2 μl of 0.04% EB solution was injected into the corpus callosum. Control animals received an equi-volume injection of pyrogen-free saline. Delivery of the gliotoxin or saline was performed at a rate of 1 μl/min after which the syringe was left in place for an extra 4 min to allow the complete diffusion of the injected solution.

### Hormonal Treatment

Hormonal treatment started 2 h post-surgery. The treatment regimen consisted of two daily subcutaneous injections of either ADIOL (dissolved in 90% sesame oil and 10% ethanol), or the vehicle alone. Rats were randomly assigned to receive the treatment for periods of 2 days (acute inflammation), 7 days (peak of demyelination) and 14 days post-surgery (beginning of remyelination; Levine and Reynolds, 1999; Kalakh and Mouihate, 2015), after which they were deeply anesthetized and sacrificed.

### Tissue Collection

Animals were anesthetized using urethane (1.5 mg/Kg, i.p.) after which transcardial perfusion was performed using ice-cold phosphate-buffered saline (PBS). For immunofluorescence studies, the brains were collected, post-fixed with a solution of 10% neutral-buffered formalin, and embedded with paraffin (Sigma Aldrich, St. Louis, MO, USA). For western blot experiments, another set of animals were subjected to demyelination and used to collect the affected area of the corpus callosum by cutting the brain coronally around the cortical landmark of the syringe entry into the brain. An area of ~2 × 2 mm of fresh corpus callosum was collected, snap-frozen in liquid nitrogen, and stored in −80°C freezer for later use as previously described (Kalakh and Mouihate, 2015).

### Immunofluorescence

Brains embedded in paraffin blocks were sectioned at a thickness of 5 μm at the level of the lesion center and mounted on slides for immunofluorescent staining as previously described (Kalakh and Mouihate, 2015). Briefly, the brain sections were deparaffinized using xylene and rehydrated through a series of ethanol solutions. Antigen retrieval was performed by boiling the sections in sodium citrate solution (10 mM) at a pH of 6.0 for 10 min. Microglia were detected using a polyclonal rabbit antibody against ionized calcium binding adapter molecule 1 (Iba1; 1:2000; Wako Chemicals, USA, Inc., Richmond, VA, USA). Microglial cells were categorized into four types (1–4) as follows: type 1 microglia have small cell perikaryon with long processes; type 2 microglia have relatively larger perikaryon with short processes; type 3 microglia have irregularly shaped and enlarged cell body with very few short processes; and type 4 microglia show an ameboid shape with no apparent processes. Type 4 microglia are morphologically similar to M1 microglia, while M2 microglia is akin to type 2 (Giulian, 1987; Zhang et al., 2014). Neuronal axons were detected using a polyclonal goat anti-medium neurofilament (NF) antibody (1:1000; Santa Cruz Biotechnology, Santa Cruz, CA, USA). The myelin sheath was detected using a mouse monoclonal antibody against myelin basic protein (MBP; 1:2000, Calbiochem, Billerica, MA, USA). The following day, brain sections were washed with PBS solution 3× for 10 min each and incubated with appropriate polyclonal donkey secondary antibodies anti-mouse, anti-rabbit, or anti-goat tagged with either Alexa 488 or Alexa 555 (1:1000) for 2 h at room temperature. The secondary antibodies were then washed off with PBS (3X, 10 min each). Brain sections were then mounted with a mounting medium and covered with a coverslip.

Images of the immunofluorescently stained sections were observed using Zeiss LSM 700 confocal microscope (Carl Zeiss, Göttingen, Germany). Images of areas of interest were acquired using AxioVision software (Carl Zeiss, Gottingen, Germany) with 40× or 63× objectives. For the analysis of NF staining, images of right and left edges of the lesion were acquired from three consecutive sections originating from 4 to 10 different rats. The density of NF^+^ fibers was analyzed by randomly selecting three different areas of each acquired image. The selected areas were binarized and the area covered by NF^+^ fibers was measured as percentage of the total area as previously described (Kalakh and Mouihate, 2015). For axonal spheroid analysis, each spheroid appearing in the field was circled and its area was measured using ImageJ software (Schneider et al., 2012). A total of ~12150 axonal spheroids were analyzed for all different treatment time points. For microglial cell count, images from the center of the lesion were acquired. Three consecutive brain sections from four to six different rats were used for counting using the cell counter macro in ImageJ software. All the above analyses were performed by an observer blind to the treatment received by each rat.

### Transmission Electron Microscopy

Transmission electron microscopy (TEM) was performed as previously described (Kalakh and Mouihate, 2015). Seven days post-demyelination, rats given either ADIOL or vehicle (EB-O; *n* = 5, EB-A; *n* = 5) were anesthetized using urethane (1.5 mg/Kg, i.p.) and perfused with ice-cold PBS. The injected area was collected by cutting the brain sagittally around the needle track mark under a dissecting microscope. The collected tissue was then post-fixed in 3% glutaraldehyde for 3 h after which they were post-fixed for 2 h in 1% osmium tetroxide. The tissue was then dehydrated using increasing concentrations of ethanol and embedded with epoxy-resin. Semi-thin sections (0.5 μm) were obtained using a glass knife mounted on an ultramicrotome. The semi-thin sections were stained with 1% toluidine blue and observed under light microscope (Zeiss Axio Observer A1) to ensure the presence of the lesion in the collected tissue. Tissue blocks were then cut in ultrathin sections (100 nm), mounted on copper grids, and stained with uranyl acetate and lead citrate for TEM observation as previously described. Images were acquired using 10,000× objective (JEOL’s JEM-1200 EXII Scanning Transmission Electron Microscope, Tokyo, Japan).

### Western Blot

Protein extraction from brain tissue was performed as previously described (Mouihate et al., 2004; Kalakh and Mouihate, 2015). Briefly, the brain tissue was homogenized in a lysis buffer (MOPS, 20 mM; KCl, 150 mM; Mg Acetate, 4.5 mM; Triton X, 1%) containing protease inhibitor cocktail tablet (Roche Applied Science, Mannheim, Germany) and centrifuged at 12,000 g for 15 min at 4°C. The supernatant containing the proteins was then collected and protein levels were assayed using bicinchoninic acid protein assay (Pierce Chemical Co., Rockford, IL, USA).

Proteins (60 μg per well) were separated using 12% SDS PAGE, transferred into a nitrocellulose membrane, and then incubated with primary mouse monoclonal antibodies anti-inducible nitric oxide synthase (iNOS; M1 microglia, 1:1000, BD Biosciences, CA, USA) or anti-arginase-1 (arg-1; M2 microglia, 1:1000, BD Biosciences, CA, USA). The following day, the membranes were washed and incubated with a secondary horseradish-peroxidase conjugated donkey anti-mouse antibody (1:2000, Santa Cruz Biotechnology, Santa Cruz, CA, USA) at room temperature for 2 h. A chemiluminescent substrate (ECL kit; GE Healthcare, UK) was applied to the membrane and proteins were detected using a Kodak X-Omat film (Eastman Kodak, New York, NY, USA). The membranes were then “stripped” off the first set of primary and secondary antibodies using β-mercaptoethanol (Sigma Aldrich, St. Louis, MO, USA) and re-incubated with primary rabbit antibody anti-actin (a house keeping protein; 1:10, 000, Sigma Aldrich, St. Louis, MO, USA). Actin protein was detected as described earlier.

Semi-quantitative estimation of protein levels was performed using ImageJ software. The optical density (OD) profile was obtained for each protein band. The area under the curve (AUC) of the OD profile was used to estimate the protein levels in each sample. The ratio of AUC values of iNOS/actin or arg-1/actin was determined and used for comparison between experimental groups.

### Statistics

Immunofluorescence data of axonal damage and western blot were compared using unpaired student’s *t* test. Data for percentage area of total NF were compared using ANOVA followed by Newman Keuls *post hoc* test. Analysis of the frequency distribution of axonal spheroids was performed using Kolmogorov–Smirnov test (K-S test). Analysis of microglial activation using immunofluorescence was performed using two-way ANOVA followed by Bonferroni *post hoc* test. A *p* value of less than 0.05 was considered statistically significant. All data are presented as mean ± SEM.

## Results

### ADIOL Reduced Axonal Damage at the Acute Stage of Demyelination

**Figure 1:**
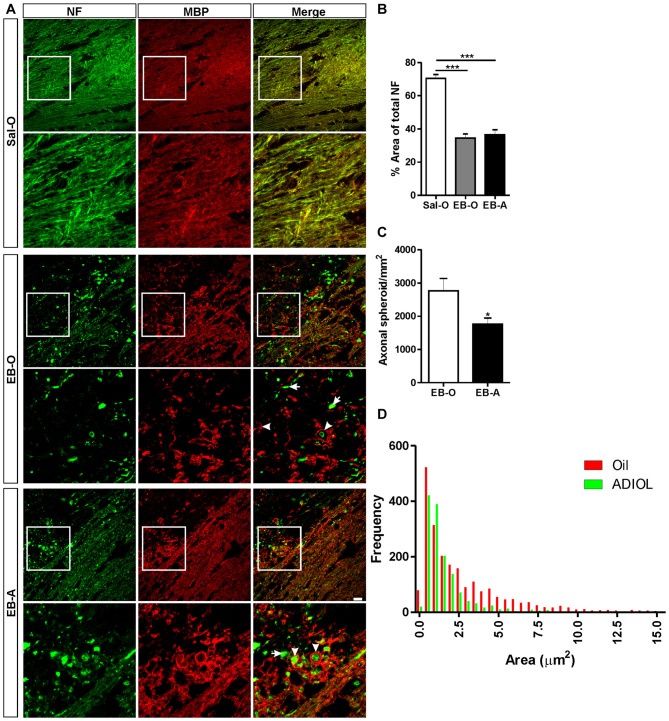
**Effect of androstenediol (ADIOL) on axonal damage 2 days post-ethidium bromide (EB)-induced demyelination. (A)** Immunofluorescent staining of neurofilament (NF, green) and myelin basic protein (MBP, red) in the corpus callosum of Sal-O, EB-O or EB-A rat groups. Injection of EB into the corpus callosum (EB-O panel) decreased the density of NF^+^ fibers when compared to that seen in saline-injected group (Sal-O panel). Systemic injection of ADIOL increased the density of NF^+^ fibers in the vicinity of the demyelinated corpus callosum (EB-A panel) compared to the one seen in EB-O rat group. At the edge of the demyelination lesion, many of NF^+^ axons in EB-O rat group either lacked myelin (arrows) or had a detached myelin sheath (arrowheads). In contrast, the axons of EB-A rat group had more compact myelin sheaths (arrowheads). **(B)** Bar graph shows the percentage area covered by NF^+^ fibers in the three rat groups (Sal-O: *n* = 4, EB-O: *n* = 4, EB-A: *n* = 6). EB injection induced a significant decrease in the area covered by NF^+^ fibers when compared to NF^+^ density in saline-injected animals (*p* < 0.001). This decreased axonal density was not affected by systemic injection of ADIOL. **(C)** Bar graph presents the number of axonal spheroids in the vicinity of the demyelination lesion in EB-O and EB-A groups. ADIOL significantly reduced the number of axonal spheroids compared to oil-treated animals (EB-O: *n* = 4, EB-A: *n* = 6, *p* < 0.05). **(D)** Histogram shows the frequency distribution of axonal spheroids according to their sizes. The majority of axonal spheroids had a size less than 2.5 μm^2^ in both EB-O and EB-A rat groups. *p < 0.05, ***p < 0.001. All data are presented as mean ± SEM. Scale bar = 50 μm.

A prominent axonal damage was evident in the vicinity of the corpus callosum 2 days post-demyelination insult as a result of EB injection. Signs of axonopathy included multiple axonal transections and swellings indicative of disrupted axonal cytoskeleton (Figure 1A middle panel, arrows). These signs were not present in saline-injected corpus callosum, which showed a healthy pattern of myelinated axons (Figure 1A upper panel). To assess the extent of axonal loss in the demyelinated corpus callosum, we measured the percentage area covered by NF^+^ fibers at both edges of the lesion. Compared to saline-injected corpus callosum, EB-injected corpus callosum showed a significant reduction in the density of NF^+^ fibers (Sal-O; *n* = 4, EB-O; *n* = 4, *p* < 0.05; Figure 1A upper panel, Figure 1B). However, there was no difference in the area fraction covered by NF^+^ fibers between oil-treated and ADIOL-treated groups (EB-O; *n* = 4, EB-A; *n* = 6, *p* > 0.05; Figures 1A,B). We also monitored the number of axonal spheroids per area (density) which are indicative of axonal damage (Sternberger and Sternberger, 1983). Treatment with ADIOL reduced the density of these axonal spheroids in the vicinity of the demyelination lesion when compared to those seen in oil-treated animals (EB-O; *n* = 6, EB-A; *n* = 6, *p* < 0.05; Figures 1A,C). Other pathological features were also observed such as axons with detached myelin sheath and disorganized myelin sheaths (Figure 1A middle panel, arrowheads). ADIOL-treated animals on the other hand showed a higher density of axons with healthy-looking myelin sheaths around them (Figure 1A lower panel, arrowheads).

We further measured the sizes of axonal spheroids in the vicinity of the demyelinated area of the corpus callosum and plotted their frequency distribution (Figure 1D). The majority of axonal spheroids seen at 2 days post-EB injection had sizes less than 2.5 μm^2^. Both ADIOL-treated and oil-treated animals had the highest frequency of axonal spheroids at sizes ~0.5–1 μm^2^. The large axonal spheroids ( >2.5 μm^2^) were less frequent in the vicinity of demyelinated area of the corpus callosum of rats injected with ADIOL when compared to those seen in vehicle-treated rats. Statistical analysis using K-S test showed a significant difference in the frequency distribution of axonal spheroids between oil-treated and ADIOL-treated groups (EB-O; *n* = 6, EB-A; *n* = 6, *p* < 0.0001, *D* = 0.252).

### ADIOL Reduced Axonal Damage at the Peak of Demyelination

**Figure 2:**
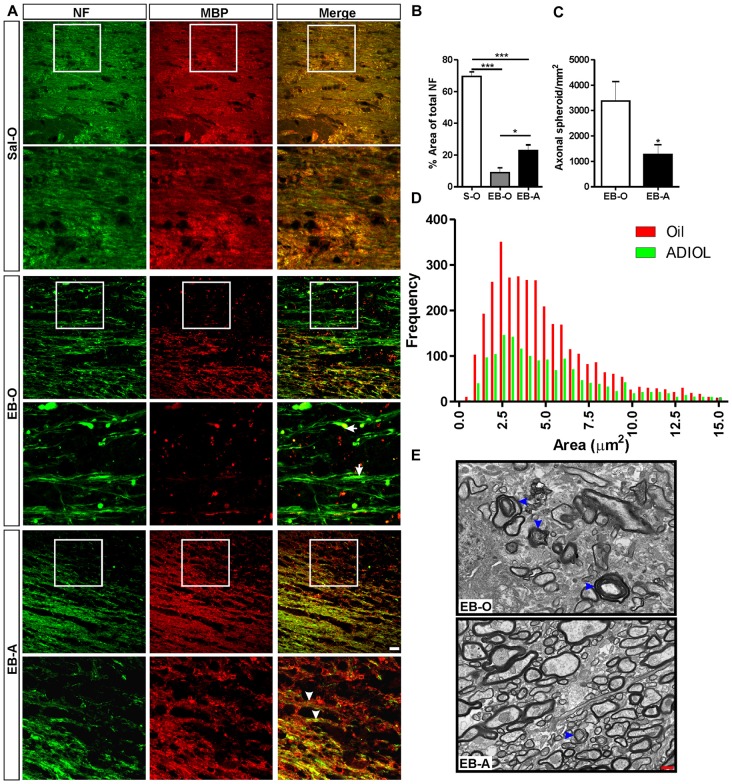
**Effect of ADIOL on axonal damage 7 days post-EB-induced demyelination. (A)** Immunofluorescent staining of NF (green) and MBP (red) in corpus callosum 7 days post-EB injection. EB injection resulted in a reduction in the density of NF^+^ fibers in the EB-O group (EB-O panel) compared to rat group given intra-corpus callosum injection of saline (Sal-O panel). Axonal spheroids indicative of axonal damage are more frequently observed in the EB-O group (EB-O panel) compared to the EB-A group (EB-A panel). **(B)** Bar graph presents the percentage area covered by NF^+^ fibers. EB injection significantly reduced the density of NF^+^ fibers in the vicinity of the demyelinated corpus callosum when compared to saline-injected animals (Sal-O: *n* = 4, EB-O: *n* = 5, EB-A: *n* = 8, *p* < 0.001). ADIOL treatment significantly increased the density of NF^+^ fibers compared to that seen in EB-O rat group (*p* < 0.05). **(C)** Bar graph shows the count of axonal spheroids in the vicinity of the EB-induced lesion. ADIOL significantly reduced the number of these spheroids compared to oil-treated animals (EB-O: *n* = 6, EB-A: *n* = 10, *p* < 0.05). **(D)** The histogram shows the frequency distribution of axonal spheroids according to their sizes. Most spheroids observed were of sizes ranging from 1 μm^2^ to 7.5 μm^2^. ADIOL reduced the frequency of these spheroids in the vicinity of corpus callosum when compared to oil-treated rats. **(E)** Transmission electron micrographs of the EB-injected corpus callosum of oil-treated and ADIOL-treated animals. Signs of myelin sheath detachment from axons are more apparent in the oil-treated animals (arrowheads in the upper micrograph) compared to those seen in ADIOL-treated ones (lower micrograph). *p < 0.05, ***p < 0.001. All data are presented as mean ± SEM. Scale bar for immunofluorescence images = 50 μm. Scale bar for transmission electron microscopy (TEM) images = 1 μm.

The integrity of axonal fibers was also assessed at the peak of demyelination which corresponds to 7 days post-EB injection. The injection of EB into the corpus callosum resulted in a decrease in the percentage area covered by NF^+^ fibers (Figure 2A middle panel) compared to the saline-injected corpus callosum (Sal-O; *n* = 4, EB-O; *n* = 5, *p* < 0.05; Figure 2A upper panel, Figure 2B). We have noticed that oil-treated animals showed multiple irregular NF^+^ spheroids (Figure 2A middle panel). Injection of ADIOL resulted in a significant increase in the density of NF^+^ fibers at the edges of the demyelination lesion compared to oil-treated animals (EB-O; *n* = 5, EB-A; *n* = 8, *p* < 0.05; Figure 2A lower panel and Figure 2B). This enhanced axonal density was accompanied by a reduction in the density of axonal spheroids in the vicinity of EB-injected corpus callosum (EB-O; *n* = 6, EB-A; *n* = 10, *p* < 0.05; Figure 2C). We also detected a significant difference in the frequency distribution of spheroids between oil-treated and ADIOL-treated groups (*p* < 0.0001, *D* = 0.087). The majority of axonal spheroids seen 7 days post-EB injection had sizes ranging from 1 μm^2^ to 7.5 μm^2^. These spheroids were less frequent in the vicinity of corpus callosum of rats given ADIOL when compared to those seen in vehicle-injected rats (Figure 2D). Because ADIOL had a stronger effect on axonal spheroids at the peak of demyelination, we explored the untrastructural change at the interface between the axons and the myelin sheet. Using TEM, we observed that there was a larger number of myelinated axons in the corpus callosum of rats given ADIOL when compared to those of rats given oil (Figure 2E). Furthermore, the detachment of myelin sheath from the axons was more frequently seen in the EB-injected corpus callosum of vehicle injected rats (Figure 2E, arrowheads).

### ADIOL’s Impact on Axonal Damage at the Initial Stage of Remyelination

**Figure 3:**
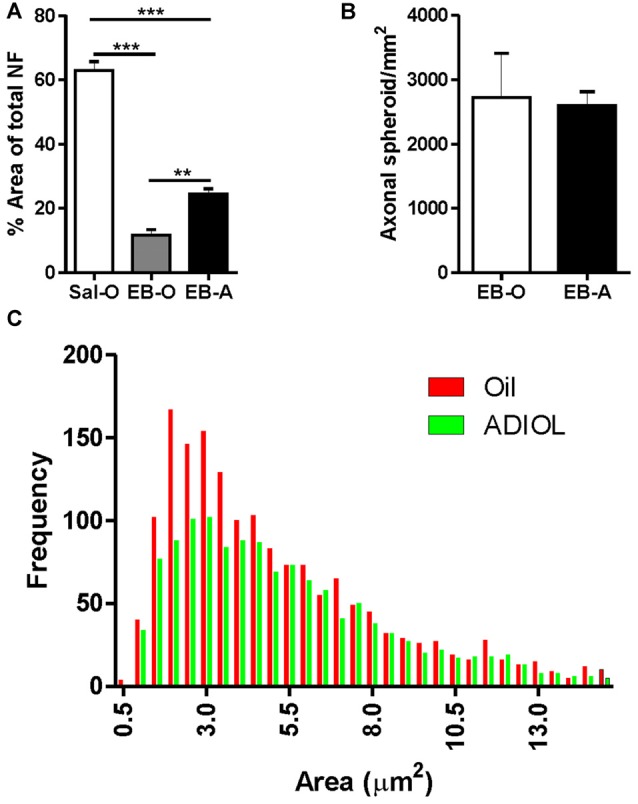
**Effect of ADIOL on axonal damage and microglial activation 14 days post-demyelination.** The bar graph in **(A)** shows the density of NF^+^ fibers in the vicinity of the demyelination lesion of the corpus callosum 14 days post-EB injection in Sal-O, EB-O and EB-A rat groups. EB injection into the corpus callosum led to a significant reduction in the density of NF^+^ fibers in the vicinity of the lesion when compared to the saline-injected group (Sal-O; *n*= 4, EB-O; *n* = 4, *p* < 0.001). In EB injected corpus callosa, the systemic injection of ADIOL induced a significant increase in the density of NF^+^ fiber compared to control animals given systemic injection of oil (*p* < 0.01; EB-O; *n* = 4, EB-A; *n* = 5, *p* < 0.01). Axonal spheroids count in the vicinity of the demyelination lesion are presented in **(B)**. Administration of ADIOL did not significantly affect the total number of axonal spheroids (EB-O; *n* = 4, EB-A; *n* = 4, *p* > 0.05). Histogram in **(C)** shows the frequency distribution of axonal spheroids according to their size. Most axonal spheroids observed were of sizes ranging from 1.5 μm^2^ to 3.5 μm^2^. Axonal spheroids of sizes less than 3.5 μm^2^ were less frequent in the vicinity of the demyelination lesion of corpus callosum of rats given ADIOL when compared to those given vehicle. **p < 0.01, ***p < 0.001. All data are presented as mean ± SEM.

The lesion was also monitored 14-days post-EB injection, a time point that corresponds to the start of the remyelination process. Compared to saline-injected corpus callosum, EB injection resulted in a reduction in the density of NF^+^ fibers at the edges of the lesion. ADIOL enhanced the density of NF^+^ fibers 14 days post-EB injection when compared to vehicle-treated animals (EB-O; *n* = 4, EB-A; *n* = 5, *p* < 0.01; Figure 3A). However, there was no significant difference in the total number of axonal spheroids at the edge of the lesion between the two experimental groups (EB-O; *n* = 4, EB-A; *n* = 4, *p* > 0.05; Figure 3B). Interestingly, axonal spheroids of relatively smaller sizes (1.5 μm^2^–3.5 μm^2^) were relatively less frequent in the EB-injected corpus callosum of rats given ADIOL when compared to those of vehicle-injected rats (*p* < 0.0001, *D* = 0.078; Figure 3C).

### ADIOL’s Impact on Microglial Activation and Polarization Post-Demyelination Insult

**Figure 4:**
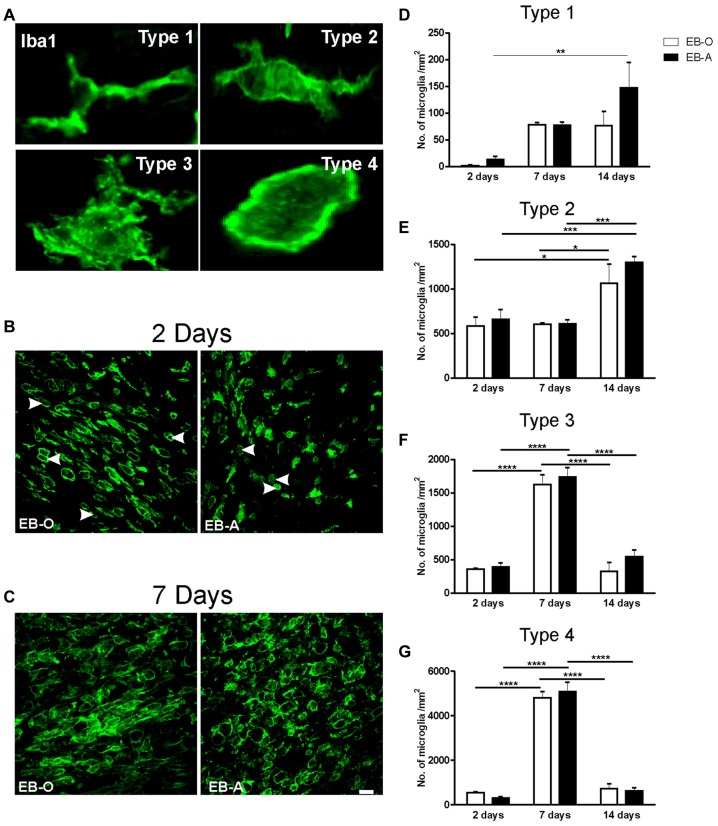
**Impact of ADIOL on microglial activation following EB injection into the corpus callosum.** Micrographs in **(A)** show the different stages of microglial activation used in the morphological analysis. Type 1 microglia have a small perikaryon and long processes. Type 2 microglia have larger cell perikaryon and shorter processes than type 1 microglia. Type 3 microglia have a large round cell body and multiple shorter branches, while type 4 has a round cell body with no apparent processes. Micrographs in **(B,C)** show microglial cells at the center of EB-injected corpus callosum at 2 days and 7 days respectively. Graph bars show cell counts of microglia of type 1 **(D)**, type 2 **(E)**, type 3 **(F)** and type 4 **(G)**, at 2, 7 and 14 days post-demyelination lesion. There was no significant effect of ADIOL on type 1 microglia at any given time point **(D)**. ADIOL administration led to a significant increase in microglial cell counts at 14 days post-EB-injection when compared to those seen at 2 days (*p* < 0.01). The number of type 2 microglia was significantly higher at 14 days post-lesion in both oil-treated (*p* < 0.05) and ADIOL-treated (*p* < 0.001) rat groups **(E)**. Both type 3 and type 4 microglia peaked at 7 days post-lesion to a significantly higher level when compared to that of 2 days and 14 days rat groups. Such increase was observed in rats given either ADIOL or oil (*p* < 0.0001; **F,G**). *p < 0.05, **p < 0.01, ***p < 0.001, **** p < 0.0001. All data are presented as mean ± SEM. Scale bar = 50 μm.

We have previously reported that ADIOL treatment resulted in a reduction in the total number of activated microglia present at the edges of EB-induced demyelination lesion (Kalakh and Mouihate, 2015). In this study, we focused our exploration on microglial activation at the center of the lesion. The perikarya of activated microglia become gradually bigger and their branches become smaller. Fully activated microglia adopt a round shape with no apparent processes. We monitored microglia at four different stages of activation at the center of the demyelination lesion (Figure 4A) and compared their cell density between vehicle-treated and ADIOL-treated animals at 2 (Figure 4B), 7 (Figure 4C) and 14 days post-demyelination insult. We observed a significant overall change in the cell density of type 1 microglia as a function of time (*F*_(2,20)_ = 8.506, *p* = 0.0021). *Post hoc* analysis showed that ADIOL administration led to a significant increase in the cell density of type 1 microglia 14 days post-demyelination lesion when compared to those microglia seen at 2 days post-demyelination lesion (*p* < 0.01; Figure 4D). However, there was no significant effect of ADIOL on the cells density of type 1 microglia at any individual time point.

We observed a significant overall change in the cell density of type 2 microglia as a function of time (*F*_(2,20)_ = 16.54, *p* < 0.0001). *Post hoc* analysis showed that there was a significant increase in the cell density of these cells in the EB-O group 14 days post-demyelination insult when compared to those seen at 2 days (*p* < 0.05) and 7 days after EB injection (*p* < 0.05; Figure 4E).

Administration of ADIOL led to a significant increase in the cell density of type 2 microglia 14 days post-demyelination when compared to that seen at 2 days (*p* < 0.001) and 7 days (*p* < 0.001) after demyelination insult. However, there was no significant effect of ADIOL on the cell density of type 2 microglia at each individual time point.

Cell density analysis of type 3 microglia showed an overall significant change over time (*F*_(2,20)_ = 90.37, *p* < 0.0001). *Post hoc* analysis revealed that there was a significant increase in the cell density of type 3 microglia 7 days post-demyelination injury when compared to 2 days (*p* < 0.0001) and 14 days (*p* < 0.0001) in both EB-O and EB-A groups (Figure 4F).

Cell density analysis of type 4 microglia showed an overall significant change over time (*F*_(2,20)_ = 259.4, *p* < 0.0001). *Post hoc* analysis showed a significant increase in the cell density of type 4 microglia at 7 days post-demyelination injury when compared to the cell density seen at either 2 days (*p* < 0.0001) or 14 days (Figure 4G; *p* < 0.0001) after demyelination lesion. However, there was no significant effect of ADIOL on the cells density of type 4 microglia at any individual time point.

**Figure 5:**
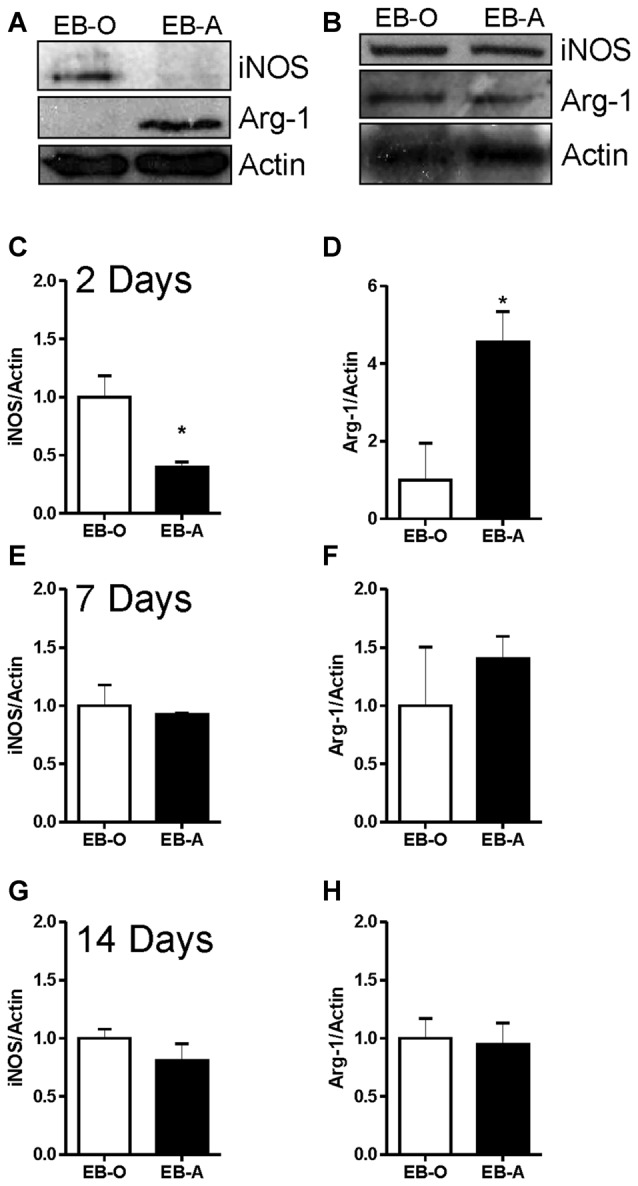
**Impact of ADIOL on microglial polarization post-EB injection.** Micrographs in **(A,B)** show inducible nitric oxide synthase (iNOS), arginase-1 (Arg-1) and actin protein expression in the corpus callosum area damaged by EB in rats given a systemic injection of either oil (EB-O) or ADIOL (EB-A) for 2 and 7 days respectively. The bar graphs present semi-quantitative analysis of western blot data at 2 day **(C,D)**, 7 days **(E,F)** and 14 days **(G,H)** after the injection of EB into the corpus callosum. Systemic injection of ADIOL for 2 days resulted in a significant reduction in the expression level of iNOS **(A,C)** when compared to its corresponding levels in EB-O rat group (EB-O; *n* = 4, EB-A; *n* = 4, *p* < 0.05). ADIOL injection for 2 days led to a significant increase in the expression level of Arg-1 protein **(A,D**) when compared to that seen in EB-O rat group (EB-O; *n* = 4, EB-A; *n* = 4, *p* < 0.05). *p < 0.05 at 7 days post-EB injection, the expression levels of iNOS **(B,E)** or Arg-1 **(B,F)** proteins were not affected by systemic injection of ADIOL (EB-O; *n* = 4, EB-A; *n* = 4, *p* > 0.05). Similarly, at 14 days after EB injection the expression levels of iNOS **(G)** or Arg-1 **(H)** proteins were not affected by systemic injection of ADIOL (EB-O; *n* = 3, EB-A; *n* = 3, *p* > 0.05). *p < 0.05. All data are presented as mean ± SEM.

We also assessed whether ADIOL treatment affects microglial polarization into either M1 or M2 phenotypes by monitoring the expression levels of iNOS (M1) and arg-1 (M2). Two days post-demyelination lesion, the expression level of iNOS was significantly reduced in the EB-injected area of the corpus callosum of rats given ADIOL when compared to those injected with the vehicle (EB-O; *n* = 4, EB-A; *n* = 4, *p* < 0.05; Figures 5A,C). Injection of ADIOL also led to an increase in the expression levels of M2 marker arg-1 (EB-O; *n* = 4, EB-A; *n* = 4, *p* < 0.05; Figures 5A,D). In addition, ADIOL did not affect the expression of either iNOS expression (M1 marker) or arg-1 expression (M2 marker) at either 7 days (EB-O; *n* = 4, EB-A; *n* = 4, *p* > 0.05; Figures 5B,E,F) or 14 days post-demyelination insult (EB-O; *n* = 3, EB-A; *n* = 3, *p* > 0.05; Figures 5G,H).

## Discussion

We have previously shown that ADIOL dampened EB-induced demyelination lesion, promoted the proliferation and maturation of oligodendrocyte progenitor cells in the demyelinated area, and enhanced the phosphorylation of MBP. These processes are all essential for a successful remyelination process (Kalakh and Mouihate, 2015). Here, we focused on ADIOL’s neuroprotective properties on axonal integrity following demyelination by exploring its impact on the inflammatory milieu triggered by the demyelination insult. In this study, we have made a number of novel observations regarding the protective effect of the neuroactive steroid ADIOL on axonal integrity following a localized demyelination lesion in the corpus callosum: (1) systemic administration of ADIOL reduced axonal abnormalities (i.e., axonal spheroids) following EB-induced focal demyelination in the corpus callosum; and (2) axonal protection was associated with dampened inflammatory response that was shifted towards the neuroprotective M2 phenotype during the acute phase.

To our knowledge, this is the first study to explore how ADIOL affects axonal recovery following focal demyelination. Previous studies have assessed the promyelinating effect of ADIOL were performed using experimental autoimmune encephalomyelitis (EAE), an experimental model with an overt activation of the immune system (Nicoletti et al., 2010; Saijo et al., 2011; Hanna et al., 2015). In this study, we induced demyelination within a small area of the corpus callosum to minimize the contribution of systemic immune response in the recovery process. This experimental model allows the investigation of ADIOL’s effect on the local inflammatory response and how it affects axonal recovery following demyelination. Furthermore, the time course for the acute phase (2 days post-EB injection) and peak of demyelination (7 days post-EB injection) as well as the starting time for the remyelination (14 days post-EB injection) has been well characterized in this experimental model (Levine and Reynolds, 1999; Gregg et al., 2007; Kalakh and Mouihate, 2015, 2016).

### Axonal Damage Following Demyelination

Axonal damage is a pathological sign of a number of neurodegenerative conditions including MS (Trapp and Stys, 2009; Haines et al., 2011), stroke and brain/spinal traumatic injuries (Czeiter et al., 2008; Hinman, 2014). Postmortem studies on brains of MS patients showed extensive axonal irregularities and transections in disease-active areas of the brain (Trapp and Stys, 2009; Nave, 2010b). The extent of axonal damage is a major determinant of disease severity (Lingor et al., 2012). In the present study we have assessed the condition of axonal damage in the vicinity of the demyelination lesion at 2, 7 and 14 days post-EB injection. Our data showed that EB injection into the corpus callosum reduces the density of axons present at the edge of the lesion from ~70% in the control animals to ~30% by the second day post-demyelination insult. This percentage was further reduced to as low as ~8% by the seventh day post-lesion. A spontaneous recovery in axonal density was seen 14 days post-lesion.

Several line of evidence suggests that ADIOL exerted an axono-protective effect. Indeed ADIOL increased the density of axons at the edge of the demyelination lesion by the seventh day of treatment from ~10% in oil-treated animals to ~20%. This recovery of axons was further increased by day 14 post-demyelination insult. Furthermore, ADIOL reduced the number of axonal spheroids present at the vicinity of demyelinated area by 2 and 7 days post-gliotoxin injection. At the ultrastructural level, administration of ADIOL enhanced the percentage of remyelinated axons in the vicinity of the lesion as we have previously shown (Kalakh and Mouihate, 2015). In addition, degenerating axons were less frequently observed in these animals. ADIOL also decreased the g-ratio of remyelinated axons, which indicates better recovery following demyelination (Kalakh and Mouihate, 2015).

### ADIOL Effect on Inflammation-Induced Axonal Damage

Axonal damage is largely influenced by the inflammatory response associated with demyelination (Lingor et al., 2012). Understanding how inflammation affects demyelinated axons can help in developing treatments to overcome demyelination-associated axonopathy and therefore reduce disease severity. The environment of EB-induced demyelination is very complex and includes the damaged cells, injured axons, myelin fragments remaining from demyelinated axons and reactive glia (microglia, astrocytes; Horn et al., 2008). Using immunofluorescence and electron microscopy, we observed the presence of multiple axonal transections, axonal spheroids, empty myelin sheaths, and disintegrated and de-compacted myelin sheaths. The inflammatory cascade is initiated in response to this damage in which microglia are crucial players (Levine and Reynolds, 1999). We observed a moderate microglial activation by 2 days post-lesion which peaked 7 days post-lesion. Microglial activation is required for clearing out cellular debris and myelin fragments in the lesion area. This protective effect of microglia appear to create conducive environment for the remyelination process (Pohl et al., 2011; Lampron et al., 2015). We have classified activated microglia based on their morphology into four types (described earlier). At 2 days post-demyelination insult, a large proportion of microglia adopted activated morphologies (types 2, 3 and 4) indicating the high responsiveness of these cells to the lesion during the acute phase. Such activation of microglia was stronger on the 7th day post-demyelination insult, especially for type 3 and type 4 microglia. Two weeks post-EB-lesion, the cell density of these activated microglia was significantly reduced to levels seen during the acute phase (2 days). This observation is in line with previously published data which established that the maximal brain inflammation occurs 7 days post-EB-induced demyelination insult, while the remyelination process is initiated spontaneously at around 14 days post-EB injection (Levine and Reynolds, 1999). We did not observe a significant effect of ADIOL on the cell density of activated microglia at any given time point. However, ADIOL administration for 14 days led to a slightly but a significantly increase in the cell density of type 1 microglia. Because morphologically this microglial cell type is assimilated with a “resting” state, it is possible that ADIOL enhances the process of remyelination (Kalakh and Mouihate, 2015) by modulating the activation level of a subset of microglia. Future studies will explore the intracellular mechanisms underlying the effect(s) of ADIOL on microglia during the remyelination phase.

One incessant question is whether peripheral macrophages contribute to the observed Iba1+ cells, we traditionally attribute to resident microglia. Owing to their morphologies and the existence of the remnant of processes in type 2 and 3 microglial types, it is unlikely that these two cell types are derived from peripheral macrophages, as the peripherally derived macrophages have been shown to be spherical in shape when they invade an affected tissue (Greenhalgh and David, 2014). However, what we have considered as type 4 microglia adopt a round shape that is undistinguished from peripheral macrophages. Thus there is a possibility that these cells are peripheral macrophages. However, there are indications that the early microglias are the major contributors to brain inflammation during the acute phase (less than 3 days; Horn et al., 2008; Olah et al., 2012).

In addition to the classification of microglia based on their morphological changes, activated microglia are also categorized into either M1 and M2 phenotypes. These microglia can produce either pro-inflammatory cytokines such as IL-1ß and TNF-α (M1 microglia), or anti-inflammatory cytokines such as IL-10 and TGF-ß (M2 microglia; Neumann et al., 2009). Axonal damage is worsened by proinflammatory cytokines produced by M1 microglia. Regulatory factors produced by M2 microglia, on the other hand, appear to play a neuroprotective for axons following demyelination (Cherry et al., 2014). Therefore, the dynamics of M1 and M2 microglial response are important for axonal recovery following demyelination. In our experiment, we found that EB injection into the corpus callosum resulted, at least during the acute phase, in enhanced expression of the M1 marker iNOS and decreased expression of the M2 marker Arg-1. As previously mentioned, ameboid-shaped microglia are likely of M1 phenotype (Zhang et al., 2014). Thus, it appears that ADIOL reduces M1 phenotype. Such inflammatory milieu is likely setting the stage for the extensive axonal damage at later time points. This M1 bias was significantly reverted when the rats were given systemic injection of ADIOL. This ADIOL-induced change in microglial bias towards M2 types strongly suggests that ADIOL promotes a shift in microglial activation, especially during the early phase of demyelination insult. Such microglial shift was lost when microglia were assessed at later stages of the demyelination process (7 and 14 days post-demyelination insult). It is noteworthy that we conducted a series of immunostaining to determine the cellular distribution of M1 and M2 markers in the demyelination lesion area. In our hands, the available antibodies against iNOS and arg-1 did not show a convincing staining at the site of the lesion of the rat corpus callosum (data not shown). As mentioned above, it is possible that the early stage of polarization is driven, at least in part, by peripheral macrophages. However, this possibility is less likely as the early phase of neuroinflammation is mainly driven by resident microglia (Horn et al., 2008; Olah et al., 2012; Greenhalgh and David, 2014). Therefore, it is possible that microglial polarization observed at 2 days post-lesion is driven mainly by resident microglia, while at later stages (7 or 14 days post-demyelination insult), the robust infiltration of peripheral macrophages does not allow the detection of clear microglial phenotype. These findings are in line with previous studies which have shown that pretreatment with ADIOL (2 days before lesion) reduced the levels of iNOS and the proinflammatory cytokines TNF-α and IL-6 in the striatum in an animal model of Huntington’s disease (Hanna et al., 2015). ADIOL was also shown to modulate inflammation towards Th2 response and increase the level of the anti-inflammatory cytokine IL-4 in EAE, an autoimmune model of demyelination (Auci et al., 2005).

## Conclusion

We give the first evidence that ADIOL reduces signs of axonopathy following EB-induced focal demyelination. Our present and previously published work suggests that ADIOL is endowed with promyelinating and neuroprotective effects. ADIOL appears to exert these beneficial effects by directing microglial activation towards a regulatory pathway, at least during the acute phase. The data presented in this study opens new research avenues for the potential therapeutic effect of ADIOL in the reduction of demyelination-induced axonopathy.

## Author Contributions

AM designed the experiments and supervised the research project. SK conducted the experiments. AM and SK analyzed the data and co-wrote the manuscript.

## Conflict of Interest Statement

The authors declare that the research was conducted in the absence of any commercial or financial relationships that could be construed as a potential conflict of interest.
